# Novel Coronavirus Outbreak and Career Development: A Narrative Approach Into the Meaning for Italian University Graduates

**DOI:** 10.3389/fpsyg.2020.02255

**Published:** 2020-10-22

**Authors:** Anna Parola

**Affiliations:** Department of Humanities, University of Naples Federico II, Naples, Italy

**Keywords:** COVID-19, young adult, mental health, unemployment, school-to-work transition, narrative approach, cluster analysis

## Abstract

**Introduction:**

In times of economic crisis, the literature shows that young people have always been in the high-risk category. The COVID-19 outbreak and the consequence on the economic level have increased the sense of uncertainty and precariousness experienced by young people. The current scenario has forced young people at the school-to-work transition point to re-think their career plans. Although the difficulties of the school-to-work transition already lead to distress and mental health problems in young people, the slowdown imposed by the coronavirus could add up to these difficulties. The present study aimed to explore the process of career development and career planning in the coronavirus era. Twenty Italian university graduates were involved.

**Methods:**

A quantitative measure was used to evaluate the affective (positive/negative) experience. A narrative prompt was used to understand the individual dimensions of career planning. Cluster analysis was carried out by an unsupervised ascendant hierarchical method to explore the themes of the narration.

**Results:**

Italian young adults have tended to experience negative affects in the recent weeks of quarantine. The themes highlighted in the narratives showed that Italian young adults experience feelings of fear, uncertainty, and anxiety about the post-pandemic future.

**Conclusion:**

The results appear as a starting point to re-think possible interventions for this group post-lockdown and post-pandemic.

## Introduction

The current coronavirus disease (COVID-19) has had a massive impact on the people in the world and on many dimensions of life. One of these concerns the economic fallout of the crisis on the people, young adults in particular. In times of economic and financial crisis, young people have always been in the category at risk. For example, after the 2008 economic crisis the NEET phenomenon (young not engaged in Education, Employment or Training) into EU-28 (EU NEET-rate, 28.8%, Eurostat, 2019) came to light. The latest report of the International Labor Organization (2020) highlights that young people represent the most vulnerable group when it comes to the social and economic impact of the virus pandemic. According to the International Labor Organization (2020), the COVID-19 economic crisis with its vast increases in unemployment may result from significant exclusion of young people from the labor market.

Currently, the conditions imposed for the COVID-19 outbreak may increase the sense of uncertainty and precariousness experienced by young people.

The novel coronavirus has forced young people in school-to-transition points to re-think their career plans. From a theoretical point of view, individuals have an active role in the construction of their career paths. Consistent with life-span developmental psychology (Baltes et al., 1980; Lerner, 1982; Lerner and Tubman, 1991; Bates et al., 1998; Bynner and Parsons, 2002), career development constitutes a life-long process from childhood (e.g., Magnuson and Starr, 2000; Hartung et al., 2005, 2008; Watson and McMahon, 2008; Ferrari et al., 2015; Bakshi, 2017) through adolescence (e.g., Blustein, 1997; Skorikov and Vondrecek, 2007; Skorikov and Vondracek, 2011), adulthood (e.g., Lea and Leibowitz, 1992; Vondracek and Kawasaki, 1995), and old age (e.g., Bohlmann et al., 2018) affected by both personal and contextual factors.

Several studies have shown possible intrinsic dimensions (e.g., personality, Rossier, 2015; cognitive style, Rogers et al., 2008; goal-orientation, Grant and Dweck, 2003; career adaptability, Rottinghaus et al., 2005; Savickas and Porfeli, 2012; identity, Kunnen, 2013; personal interest, Lent et al., 2010; Nyamwange, 2016; self-efficacy, Howard et al., 2009; Fan et al., 2014; Guan et al., 2015; Hui and Lent, 2018), and extrinsic influences (social support, Seibert et al., 2001; Kracke, 2002; Wiesenberg and Aghakhani, 2007; peers, Steinberg et al., 1992; parents, Paa and McWhirter, 2000; Halpern, 2005; Greenhaus and Powell, 2006; Schultheiss, 2006; Marcionetti and Rossier, 2016; teachers and educators, Howard et al., 2009; Gokuladas, 2010; Cheung et al., 2013; Cheung and Arnold, 2014) that affect the career development process in life span. Moreover, other studies have highlighted that cultural aspects have an impact on career choices (Mau, 2000; Caldera et al., 2003; Wambu et al., 2017; Akosah-Twumasi et al., 2018; Hui and Lent, 2018; Tao et al., 2018).

Transversally, the context in which individuals construct their careers are changing over time. Therefore, different cohorts of adolescents will experience their career outcomes differently. Indeed, the construction of career plans follows changes in the environment. Several studies show that the context of youth transitions is critically important in determining their shape and their outcomes (Baltes et al., 1980; Bynner and Parsons, 2002; Bynner, 2012; Lerner and Tubman, 1991).

In this century characterized by uncertainty and instability of the labor market, employment insecurity, and fragmented career paths (Baruch and Bozionelos, 2011), the difficulties in school-to-work transition could lead to distress (Bjarnason and Sigurdardottir, 2003; Parola and Donsì, 2018, 2019; Fusco et al., 2019; Parola et al., 2019; Stea et al., 2019), anxiety, discouragement, and maladaptive behavior (Schwartz et al., 2005; Arnett, 2007; Reifman et al., 2007). Moreover, this condition could impact on mental health (McKee-Ryan et al., 2005; Paul and Moser, 2009; Parola and Donsì, 2018; Bartelink et al., 2019), quality of life (Forma et al., 2017; Kivijärvi et al., 2019), and life satisfaction (Santilli et al., 2017).

The COVID-19 pandemic could exacerbate the school-to-work transition and add further difficulties that concern the labor market, such as the economic crisis of companies, temporary closure of offices, and the blocking of new job hires. Along with this, the unpredictability of the future, post-COVID-19, must also be considered.

Furthermore, the recent psychological literature on COVID-19 showed that young people are the highest-risk category for mental illness (Cao et al., 2020; Huang and Zhao, 2020). Studies on the psychological impact of the coronavirus in China have shown a psychological effect as moderate-to-severe, and about one-third of the population reported moderate-to-severe anxiety (Wang et al., 2020). Few recent studies on the Italian context showed that young adults have experienced internalizing and externalizing health problems during quarantine (Parola et al., 2020). Alongside this, research on previous epidemics (i.e., SARS and MERS) showed a wide range of psychosocial impacts on people during outbreaks of infection, i.e., fear of falling sick, feelings of helplessness (Hall et al., 2008; Van Bortel et al., 2016) anxiety, post-traumatic stress symptoms, and anger (for a review, Brooks et al., 2020). Moreover, several studies have highlighted significant psychiatric morbidities in non-infected younger age during the SARS epidemic (Sim et al., 2010).

The community of vocational psychology has recently broken down the problem and launched a debate on the relationship between unemployment and/or unemployment risk and health in this coronavirus era (Blustein et al., 2020). As recommended by the authors, the need arises to give a voice to young people by focusing on their experiences through qualitative and quantitative research methods. It is urgent to explore the shifts in youths’ sense of identity and their career aspirations, which may be dramatically affected by the crisis.

This study aims to understand the youth perception of how the pandemic could affect their work-transition. Therefore, the current study focuses on the construction of their career plans, the school-to-work transition, the future time perspective, and the health consequences in the coronavirus era. This is also an attempt to provide a contribution to developing psychological interventions that take into account the impacts of this situation on young people and their career plans. Indeed, counseling programs must always take into account changes in the context (Masdonati, 2019).

## Materials and Methods

### Participants and Procedure

Twenty Italian university graduates took part in this study (M_age_ = 24.4, SD_age_ = 2.04; range 22–29). The sample included six males and fourteen females. Participants were Italians from a Southern region characterized by serious youth unemployment problems (28.8%, Istat, 2019). All students lived at parental homes in the data collection procedure.

Non-probability sampling was used. In line with the explorative nature of the study, and also with the complex current historical moment, the choice of non-probability sampling makes the design for collecting data more flexible. Non-probability sampling techniques allow for drawing samples from a larger population without requiring a random selection. The specific characteristic of this sampling is the subjective judgments of the researchers that chose which units of the population to include (Henry, 1990; Tansey, 2007). Specifically, consistent with purposive sampling, young adults who would be reasonably likely to be moving into jobs were involved (Lincoln and Guba, 1985). The participants were recruited by asking guidance counselors and mentors of theses of the University of Naples Federico II.

Approval of the University Research Ethics Committee was obtained for collecting data. Due to COVID-19, students were enrolled online. Participants were informed about a complete guarantee of confidentiality and the voluntary nature of participation. Participants voluntarily accessed the online platform used for data collection. No time limit was handed out, giving freedom of expression to the participants. The respondents did not receive payment for their participation.

### Measures

For this study, mixed methods were used. A quantitative measure in the form of self-report to evaluate affective experience (positive/negative) of young people in the COVID-19 pandemic, with qualitative ones, in the form of a narrative prompt to understand the subjective dimensions of career planning experience were used.

*Positive and Negative Affect* (PANAS; Watson et al., 1988; Terraciano et al., 2003): The instrument consists of 20 self-rating items corresponding to adjectives that describe different states, feelings, and emotional experiences linked to positive (PA; 10-items; e.g., “Excited,” “Active”) and negative affects (NA; 10 items; e.g., “Nervous,” “Distressed”). Participants responded to each item on a 5-point Likert scale. Each rating seeks to measure the intensity of that specific feeling or emotion during a given timeframe for the participant from 1 (= *very slightly or not at all*) to 5 (= *extremely*). Simple amendments to the original instructions of the PANAS can be implemented to better address state fluctuations in PA and NA. In this study, participants were asked to rate their feelings “during the past few weeks.”

*Narrative Prompt* (Pizzorno et al., 2014): The narrative method (McAdams et al., 2001) was chosen to collect the career stories of participants. The narrative written prompt was designed following Pizzorno and colleagues (2014). Individuals were asked to create their career stories, recall the past, analyze the present, and anticipate the future. The questions addressed were: “Where are you in your life, and how have you arrived there? Start from whatever point you like. Were there any turning points in this story? On these occasions, what choices did you make, what difficulties did you encounter, how did you take things forward? What are your projects for the future? Now that you have told me your story, do you think the current moment could influence the realization of your plans?”

### Data Analysis

In the first step, preliminary analysis (means and standard deviations) on the quantitative data were carried out. Following Margherita and Tessitore (2019), the results of quantitative measures were used as an illustrative variable in the analysis of the interviews. Specifically, the results obtained by PANAS were dichotomized (PA and NA) according to the tendency of positive or negative affectivity of the participant and used as descriptive variables during the analysis of the interviews.

In the second step, the corpus of autobiographical narration was analyzed from data analyses of textual data (Lebart and Salem, 1994; Lebart et al., 1998) using the T-Lab software. The tools are the ones most used in health psychology (for a review, Mazzoni et al., 2018). The corpus was previously handled by customizing the dictionary through (a) lemmatization and (b) disambiguation of words. Lemmatization is the reduction of corpus words to their respective lemma. According to the linguistic issue, the entry corresponds to a lemma that defines a set of words with the same lexeme and the same grammatical category. The disambiguation allows for distinguishing the significant meanings among the different forms, i.e., the same graphic form but different meanings. Firstly, preliminary analysis of lexical richness were performed. Then, a cluster analysis (CA) through thematic analysis of elementary context was carried out by an unsupervised ascendant hierarchical method (bisecting K-means algorithm) characterized by the co-occurrence of semantic features (Karypis et al., 2000; Savaresi and Boley, 2001). The unsupervised clustering consists of the (a) construction of a data table context units x lexical units, (b) TF-IDF normalization and scaling of row vectors to unit length according to the Euclidean norm; (c) clustering through the method bisecting K-means and the measure of cosine coefficient; and (d) choice of the obtained partition and construction of a contingency table lexical units x clusters, χ^2^ test, and correspondence analysis. In this phase, the dendogram allows us to check the tree structure of the various bisections and the characteristic words of each cluster.

The tool segments narratives into elementary context units (e.c.u.) classified according to the distributions of their lemmas in terms of co-occurrences. For this study, in line with the literature (Bolasco, 1999), to guarantee the reliability of statistical computations, a minimum frequency threshold to select lemmas was set at 3. Each thematic cluster, determined by an algorithm that uses the relationship between intercluster variance and total variance, and it takes as optimal partition the one in which this relationship exceeds the threshold of 50%, consisted of a set of keywords, which were ranked according to the decreasing value of chi-square.

Through cluster analysis, it was possible to construct and explore the contents of the narrations and allows them to map the specific topics of participants (Lancia, 2004, 2008). The clustering procedures allow for a better understanding of youth discussion topics (Santelli et al., 2018; De Stefano and Santelli, 2019; Felaco and Parola, 2020). Finally, the clusters and the illustrative variables in a factorial plane graphically showed the relationship between clusters and variables. Gender and PA/NA variables were used as illustrative variables.

## Results

The preliminary analysis of PANAS showed that the NA dimension was higher than the PA dimension (M_NA_ = 3.30, SD_NA_ = 0.81; M_PA_ = 2.95, SD_PA_ = 0.64). The propensity of PA or NA dimensions showed that 65% of young people (*n* = 13) had experienced more negative affectivity in recent weeks, while 35% of young people (*n* = 7) more positive affectivity. These results were used as an illustrative variable in the analysis of the interviews.

The preliminary analysis of textual data showed that the corpus was constituted of 20 elementary contexts (e.c.), 754 lemmas, 6,249 tokens, and 744 types. In line with the propensity of PA or NA dimensions, the indexes of lexical richness showed 38.40% of the textual corpus contained the POS narrations and 61.60% of the NEG narrations.

**FIGURE 1:**
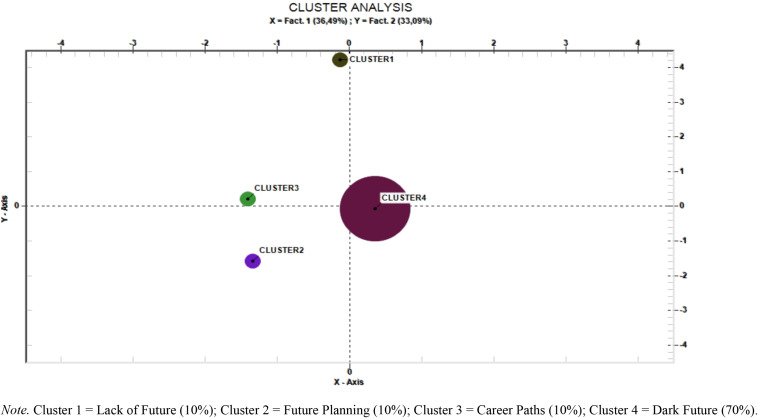
Cluster analysis. Cluster 1, Lack of Future (10%); Cluster 2, Future Planning (10%); Cluster 3, Career Paths (10%); Cluster 4, Dark Future (70%).

The thematic analysis of elementary contexts produced 4 clusters (Figure 1 and Table 1), named “Lack of Future” (10%, 2 e.c.), “Future Planning” (10%; 2 e.c.), “Career Paths” (10%; 2 e.c.), and “Dark Future” (70%; 16 e.c.).

**TABLE 1:** Clusters, associated lemmas, χ^2^ values, and significance.

**Clusters**	**Lemmas**
Cluster 1 Lack of Future (10%)	Right now (χ^2^ = 99.491; *p <* 0.001); Heavy (χ^2^ = 99.491; *p <* 0.001); Instrument (χ^2^ = 90.676; *p <* 0.001); Uncertainty (χ^2^ = 58.595; *p <* 0.001); Insecurity (χ^2^ = 48.847; *p <* 0.001); Feeling (χ^2^ = 42.574; *p <* 0.001); Restlessness (χ^2^ = 42.549; *p <* 0.001); Resignation (χ^2^ = 41.481; *p <* 0.001); Stop (χ^2^ = 32.787; *p <* 0.001); Loneliness (χ^2^ = 30.307; *p <* 0.001); Impotence (χ^2^ = 22.012; *p <* 0.001); Mood (χ^2^ = 20.012; *p <* 0.001)
Cluster 2 Future Planning (10%)	Normality (χ^2^ = 142.937; *p <* 0.001); Opportunity (χ^2^ = 37.787; *p <* 0.001); Positive (χ^2^ = 29.013; *p <* 0.001); Realization (χ^2^ = 16.685; *p <* 0.001); Plans (χ^2^ = 13.685; *p <* 0.001); Degree (χ^2^ = 10.286; *p <* 0.001); Thinking (χ^2^ = 9.935; *p* = 0.002); Projects (χ^2^ = 7.474; *p* = 0.006); Dreams (χ^2^ = 4.489; *p* = 0.034); Living (χ^2^ = 4.489; *p* = 0.034)
Cluster 3 Career Paths (10%)	Faculty (χ^2^ = 46.910 *p <* 0.001); Collocation (χ^2^ = 31.218; *p <* 0.001); Choice (χ^2^ = 16.182; *p <* 0.001); Studying (χ^2^ = 16.182; *p <* 0.001); Obtaining (χ^2^ = 7.474; *p* = 0.006); Skills (χ^2^ = 5.084; *p* = 0.024); Interests (χ^2^ = 5.084; *p* = 0.024); Labor market (χ^2^ = 5.084; *p* = 0.024); Family (χ^2^ = 5.064; *p* = 0.024)
Cluster 4 Dark Future (70%)	COVID Spread (χ^2^ = 99.491; *p <* 0.001); Dark Future (χ^2^ = 99.491; *p <* 0.001); Anxious (χ^2^ = 42.549; *p <* 0.001); Opportunity (χ^2^ = 29.013; *p <* 0.001); Epidemic (χ^2^ = 22.012; *p <* 0.001); Reaching (χ^2^ = 10.248; *p <* 0.001); Slowing down (χ^2^ = 5.084; *p* = 0.024); Difficult (χ^2^ = 5.069; *p* = 0.024); See myself (χ^2^ = 4.217; *p* = 0.024); Life (χ^2^ = 4.217; *p* = 0.024)

The first cluster, “Lack of Future,” included lemmas that refer to an uncertain future vision (lemmas “uncertainty,” “insecurity”). The lemmas “instrument,” “impotence,” and “stop” described the impasse in which young people have found themselves, without those “instruments” to deal with the current situation. In addition to it, this condition impacts the mood and sense of loneliness felt (lemmas “mood,” “loneliness”).

Examples of e.c.u.:

“The heaviest thing in this condition is to have no security and feeling like every moment you have to question yourself, without having any instrument.”

“The uncertainty of the future right now is the frequent feeling that I happen to be ridden with.”

“Stuck at home, all I do is feel restless. Unfortunately, all we can do is surrender to the evidence of a future that we cannot build because it has been taken from us.”

The second cluster, “Future Planning” described the future projects and aspirations of young people (lemmas “realization,” “dreams,” “plans”) that are entwined to a desire for “normality,” “opportunity,” and “positivity.”

Examples of e.c.u.:

“I am worried about my plans given the situation we are experiencing nowadays, but I try to be positive thinking that everything will soon return to normal, and I will be able to carry out my plans.”

“We will get back to normality and achieve what we have fought for in these years.”

The third cluster “Career Paths” offered a reflection on the transition to the labor market. This cluster described the choices that these individuals had to make in building their careers (lemmas “degree,” “studying”), also analyzing the “skills” acquired during their paths. It took into account how these skills could enhance a transition to the labor market. Moreover, the family dimension was considered (lemma “parents”) as support in the career construction process.

Examples of e.c.u.:

“I just graduated. Studying is a revelation for me, although many times, I asked myself if I was attending the right faculty. The degree, however, allowed me to acquire the proper knowledge and skills to be able to work in the area that I have chosen for myself.”

“The instrumental support of my family, but also the emotional one, allowed me to carry on my choices, graduate, and get to where I am now.”

The fourth cluster “Dark Future” presented the impact of COVID on the future, which, according to young people, has slowed down the transition to the labor market (lemma “slowdown”). The transition becomes even more “difficult” and the future “darker.” Then, in this cluster lemmas that refer to the health condition of young people emerged. Young people described distress in situations and anxiety. The presence in this cluster of the lemmas “COVID Spread” could inform on the fact that young people consider the economic consequences of the coronavirus on their future as workers.

Examples of e.c.u.:

“I am anxious about the future and seeing how the situation will be after the crisis.”

“Once we overcome COVID-19, what future will be there for us young people in the labor market?”

“The long-term effects of COVID-19 will be worse than those of the economic crisis that we are experiencing in recent years. We, young people, are always the ones who will pay the consequences.”

“Before the pandemic, I started looking for work, I even had it, a real job, of those with a proper contract. Then everything stopped, they told me they don’t know if they can hire me. They will let me know. Yes, but when? We really didn’t need this pandemic.”

“It is a very difficult period to manage emotionally. The pandemic is slowing down my plans. I can’t see after the quarantine; I can’t understand what will happen. I don’t think the epidemic will make me change my personal and professional life plans, but it has certainly made their realization more difficult.”

“This period of the pandemic has fueled even more of my fear of not being able to fulfill myself because with the economic crisis we are living with, and we will live with in the future. I believe that the working world can offer me, as well as everyone, even fewer opportunities.”

“I am anxious about the future and seeing what the situation will be after the crisis.”

The relationship between the clusters and gender variables showed that the elementary contexts associated with the female modality were present in the third cluster (15.38%) and the fourth cluster (69.23%); while the elementary contexts related to the male modality were present in cluster one (28.57%) and cluster four (71.43%).

The relationship between the cluster and the affected variables showed that the elementary contexts associated with the POS modality were present in cluster two (71.43%) and cluster three (28.58%); while the elementary contexts associated with NEG were present in cluster one (15.38%), cluster three (15.38%), and cluster four (69.23%).

## Discussion

The present study aimed to understand the experiences of the career planning of young people in the coronavirus era.

The quantitative data illustrated the current affect dimensions of young adults. Results showed that Italian young adults tended to experience negative affects in the recent weeks of quarantine. This evidence is in line with several studies that show the impact of epidemics on mental health (Brooks et al., 2020), and also with some studies that indicated higher levels of anxiety, distress, and depression in a young adults’ sample (Cao et al., 2020; Huang and Zhao, 2020). Young people can represent a high-risk category for mental illness, and this was also confirmed by recent Italian studies (Parola et al., 2020; Rossi A. et al., 2020).

The quarantine condition has imposed significant limitations, forcing young people to stay at home with their parents, limiting sports activities, and the avoidance of any contact with friends with whom they regularly experience moments of conviviality at a young age (Benedetto et al., 2018); it has allowed online relationships as the only opportunity (Faccio et al., 2019; Boursier et al., 2020). The condition of inactivity and the mandated social distance have probably triggered a state of discomfort, distress, and loneliness among young people (Rossi et al., submitted).

The participants of this study were representatives of a section of young Italians. They had completed their university studies with the achievement of their degree shortly before the lockdown period. For the young people in the school-to-work transition phase, quarantine has also imposed a limitation on the time horizon, forcing aspirations and plans for the future to be blocked. This scenario has occurred at a time of difficulty for young people in the transition from school to the labor market, which already represents a risk factor for mental health (McKee-Ryan et al., 2005; Paul and Moser, 2009), specifically in the Italian context (Parola and Donsì, 2018).

The analysis of the narrative data showed how young people are stuck in the present time, almost suspended, aware of their skills given by the years of training and university courses, but without knowing how and where to direct their strengths. Young people described distress, feelings of discomfort, and helplessness in uncertain situations in which they have no control. Results showed that young Italian adults experience psychological problems, feelings of impotence, restlessness, but also anxiety. Furthermore, these young people felt damaged by the pandemic’s potential economic fallout. These findings were strongly shown in cluster 4, which contained 70% of the youth narratives. Moreover, this cluster grouped elementary content units of young people that have experienced more negative affectivity in the weeks before the administration.

The results appear as a starting point to re-think possible interventions for this risk-group. The need for preventive interventions to support career paths during this moment of emergency seems urgent. In this sense, the narrative fosters a mediation between young people and experience (Tessitore and Margherita, 2019, 2020; Felaco and Parola, 2020; Parola and Felaco, 2020). Following Blustein et al. (2020), counseling programs must take into account the difficulties that young people will encounter in the school-to-work transition after the pandemic. Interventions must be aimed at supporting the daunting challenge of this transition and recovering from the psychological and vocational fallout of this pandemic. Even more, interventions should guarantee a positive orientation toward future vision promoting hope and optimism (Ginevra et al., 2018; Santelli et al., 2018) and enhancing the development of effective coping strategies (i.e., career adaptabilities, Savickas, 1997).

Alongside this, interventions that facilitate help-seeking for young people and improve their well-being would be desirable. The risk is that young people, even after the lockdown implementation, will find themselves even more lost in career construction. Recent studies showed that a large number of young people avoid seeking psychological help (Sareen et al., 2007; Mannarini et al., 2017a, 2018, 2020; Mannarini and Rossi, 2019; Rossi and Mannarini, 2019; Rossi Ferrario et al., 2019; Rossi Ferrario and Panzeri, 2020). In this sense, guidance and counseling activities located in universities could play a central role in supporting young people in the recovery after the pandemic. Furthermore, the request for a clinical setting becomes urgent in situations of profound fear and anxiety (Sommantico et al., 2017; Merlo, 2019a,b; Settineri et al., 2019).

The present study is not free from limitations. First of all, the bias of the non-probability sampling techniques (e.g., the selection bias) that although allows the researcher to control the selection process severely limits the generalization of the results (Flick, 2011). Secondly, the small group of participants should be increased to make more generalizable results. Although the literature on qualitative research does not indicate the determination of sample size, several studies recommend a range of 20–30 interviews for grounded research and 15–30 interviews for case studies (Marshall et al., 2013). According to the general guideline of qualitative research (Boddy, 2016) in this study the sample-size depended to the scope of the study and nature of the topic (Morse, 2000), the contact time to be spent on each research participant for career interviews (Marshall et al., 2013), and the homogeneity of the population under consideration (Trotter, 2012). Furthermore, the sample was only composed of graduates who came from the Campania region in Southern Italy. Therefore, the sample is not representative of the Italian population. Results need to be replicated in other geographical areas (northern and southern region) to provide more robust data and determine their generalizability. Thirdly, the sample is not gender-balanced, and the discussion section did not concern the gender variables. Further studies with balanced samples are needed to determine gender influence over the thematic clusters that emerged. Moreover, the study does not take into account some dimensions that could be useful for a better understanding of young experiences. Future investigations can be oriented to investigate the role of social support (Ratti et al., 2017), specifically the parental support (Balottin et al., 2017; Manna and Boursier, 2018) and love relationships (Mannarini et al., 2013, 2017b; Margherita et al., 2018).

Despite the limitations, this study contributes new knowledge about young adults’ perception of school-to-work transition in this historical moment. The current findings have several conceptual and practical implications that highlight the importance of providing tangible support to the transition from university to the world of work during this crisis. Moreover, from a methodological point of view, the study confirms the importance of the joint use of qualitative and quantitative methods in psychology. Using both a quantitative method and a narrative prompt yielded more in-depth information than either method alone would have yielded. The mixed methods have allowed, on the one hand, to quantitatively figure out the positive/negative affects related to the coronavirus through using the validated instrument; and, on the other hand, to understand the meaning given to the career paths and how coronavirus could impact on their school-to-work transition in depth, through the use of a narrative prompt.

## Data Availability Statement

The datasets presented in this article are not readily available because to ensure the privacy of the participants. Requests to access the datasets should be directed to Anna Parola.

## Ethics Statement

The studies involving human participants were reviewed and approved by the Local Ethical Committee for research in Psychology of University of Naples Federico II. The patients/participants provided their written informed consent to participate in this study.

## Author Contributions

AP contributed to the whole manuscript in each of its parts.

## Conflict of Interest

The author declares that the research was conducted in the absence of any commercial or financial relationships that could be construed as a potential conflict of interest.
